# Supplementary material for: Development and validation of self-monitoring auto-updating prognostic models of survival for hospitalized COVID-19 patients
Source: Nat Commun. 2022 Nov 10;13:6812. doi: 10.1038/s41467-022-34646-2 (PMC9648888; doi:10.1038/s41467-022-34646-2)
Supplement: Supplementary file 1 — Supplementary Information [file 41467_2022_34646_MOESM1_ESM.pdf]

Development and Validation of Self-Monitoring Auto-Updating Prognostic Models of Survival for Hospitalized COVID-19 Patients

Levy et al.

Supplementary Figures

Supplementary Figure 1. **Retrospective and prospective validation of static 7-day survival models. a** ROC and PR curves with AUC and 95% CI for the retrospective (n=1889) and prospective (n=25677; no updates) validation cohorts, **b** calibration plots for the retrospective validation cohort, **c** calibration plots for the prospective (no updates) validation cohort, and **d** decision curves for the retrospective and prospective (no updates) cohorts based on the original 7-day NOCOS, logistic regression, and XGBoost models. The blue dots on the calibration plots show the actual proportion of outcomes averaged over deciles of the predicted probabilities. The red histograms show the counts of patients that survived past 7 days binned by the predicted probabilities. The green histograms show the counts of patients that died before 7 days binned by the predicted probabilities. The diagonal black lines indicate perfect calibration. The ICIs along with their 95% CIs are reported. ROC = receiver operating characteristic; PR = precision recall; AUC = area under the ROC or PR curve; CI = confidence interval; ICI = Integrated calibration index.

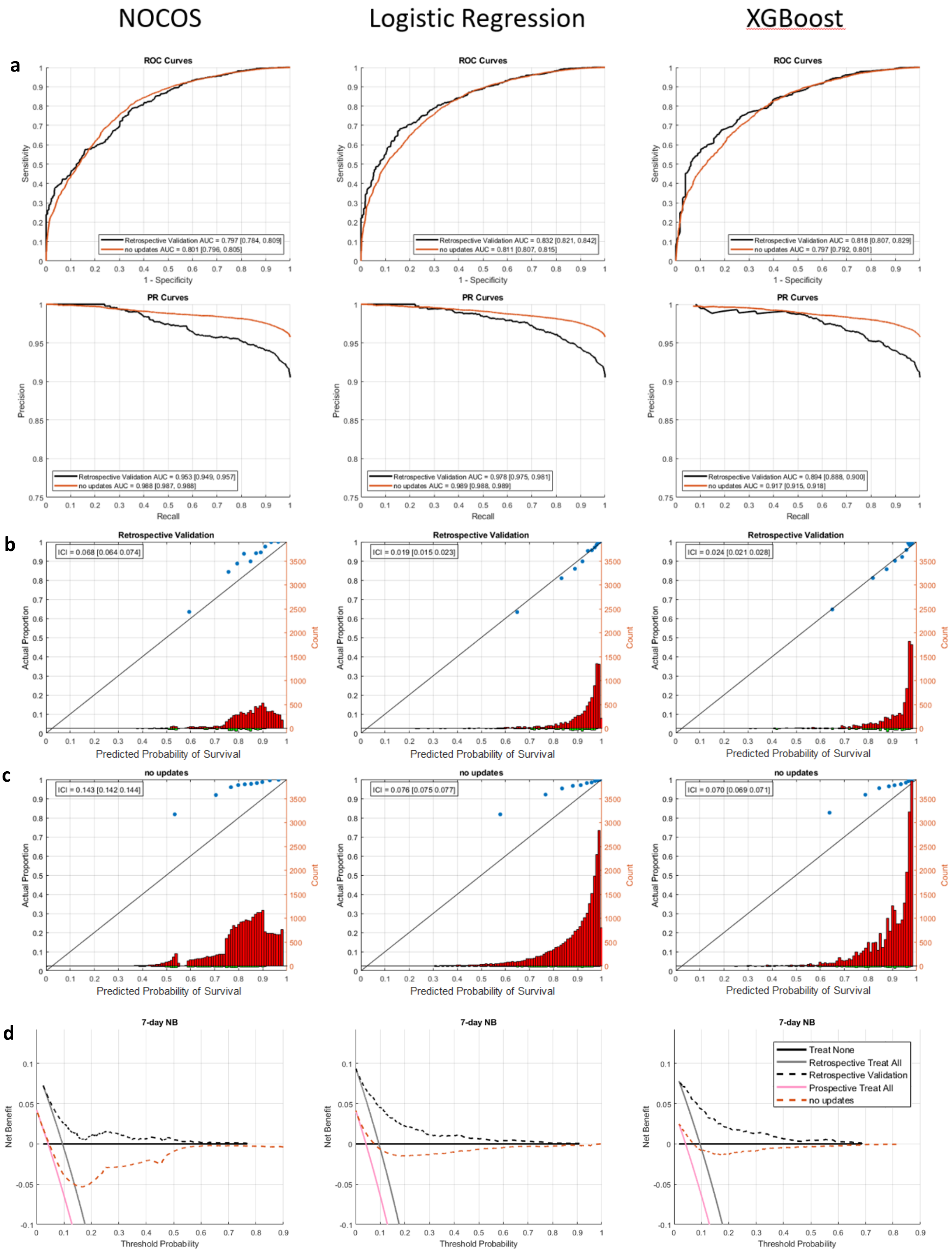

Supplementary Figure 2. **Temporal progression of performance metrics across all 7-day survival models and updating procedures.** Discrimination (AUROC) and calibration (ICI) performance metrics in a 2000-patient sliding window with a step size of 500 patients for no updates and **a** dynamically updated 7-day NOCOS, **b** logistic regression, and XGBoost models. The updating methods are listed in the legend. Updates are performed when the ICI is greater than the threshold of 0.03.

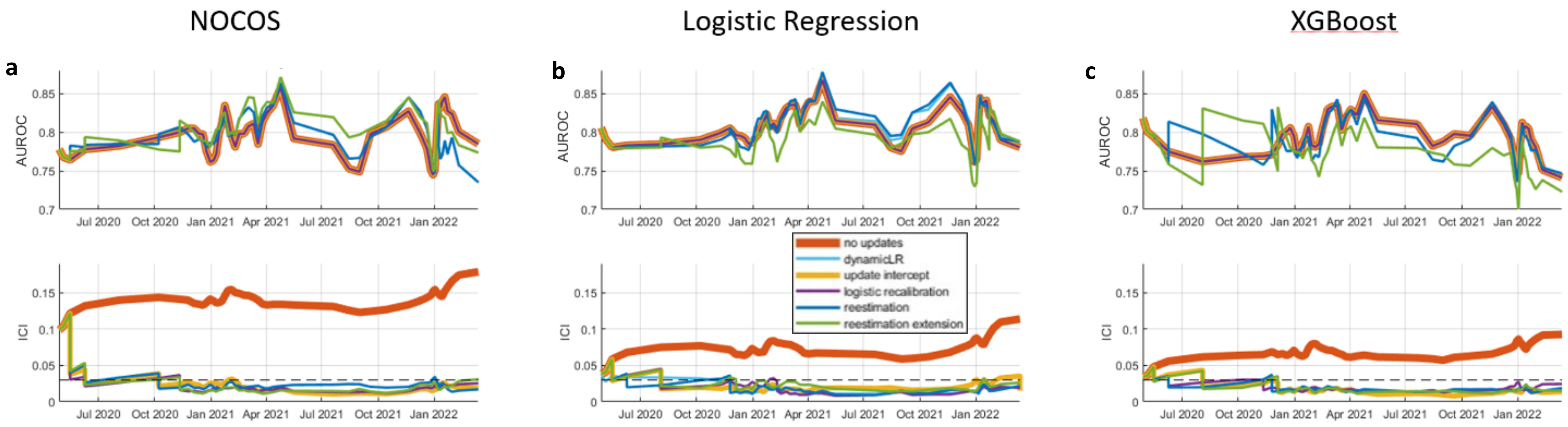

Supplementary Figure 3. **Prospective validation of all 7-day self-monitoring, auto-updating models. a** ROC and PR curves with AUC and 95% CI for the prospective (n=25677) validation cohort, and **b** calibration plots for the prospective validation cohort based on 7-day NOCOS updated using logistic recalibration, logistic regression updated using logistic recalibration, and XGBoost updated using intercept only recalibration. The blue dots on the calibration plots show the actual proportion of outcomes averaged over the deciles of the predicted probabilities. The red histograms show the counts of patients that survived past 7 days binned by the predicted probabilities. The green histograms show the counts of patients that died before 7 days binned by the predicted probabilities. The diagonal black lines indicate perfect calibration. The ICIs along with their 95% CIs are reported. c Decision curves for each model show the results for the retrospective cohort and each of the updating methods for the prospective cohort. ROC = receiver operating characteristic; PR = precision recall; AUC = area under the ROC or PR curve; CI = confidence interval; ICI = Integrated calibration index.

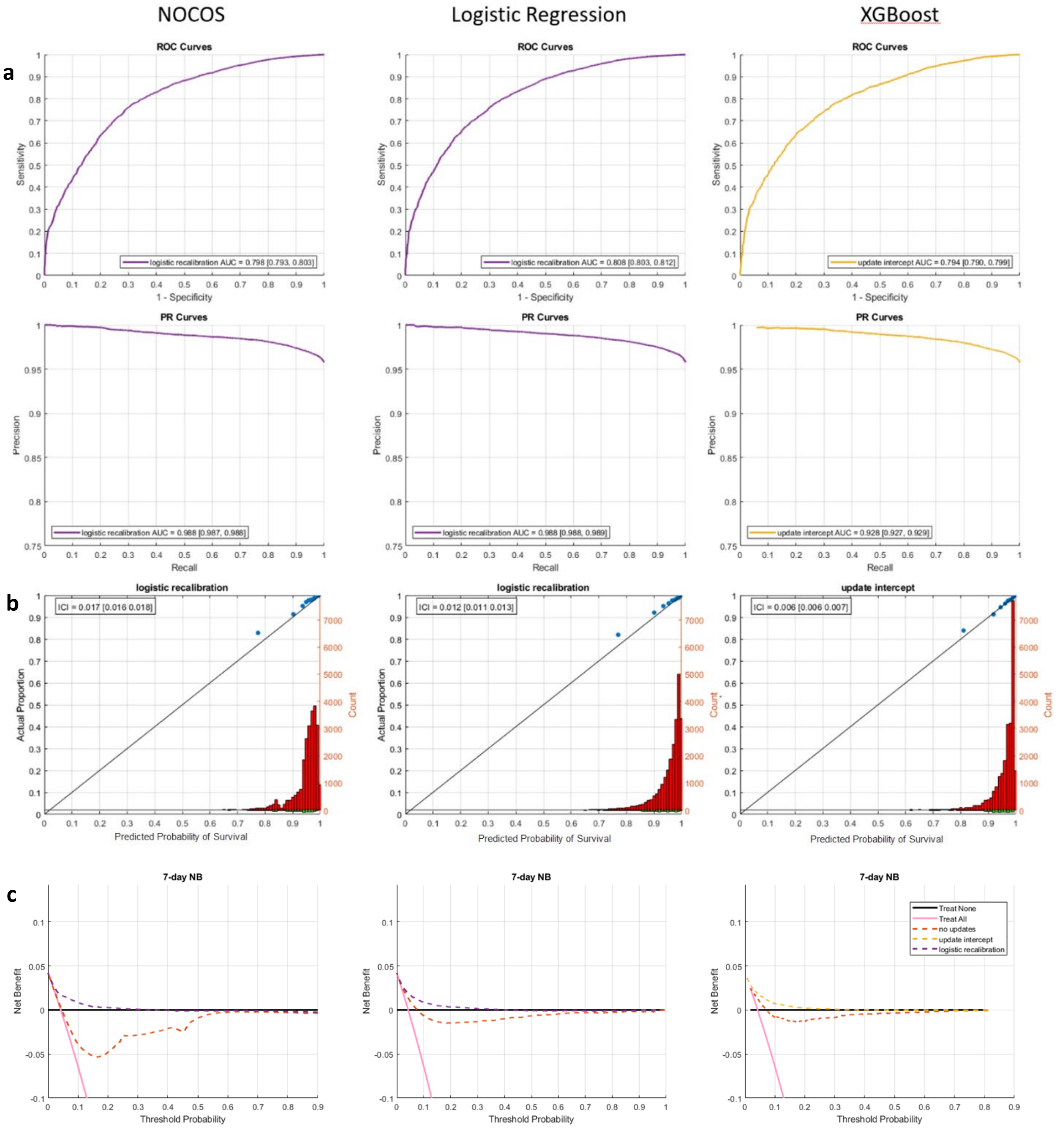

Supplementary Figure 4. **Comparison of the logistic regression coefficients updated with logistic recalibration and dynamic Bayesian logistic regression. a** the 28-day model coefficients and **b** the 7-day coefficients. While the logistic regression coefficients do not change due to logistic recalibration, we plot the combined effect of the logistic calibration coefficients and the logistic regression coefficients.

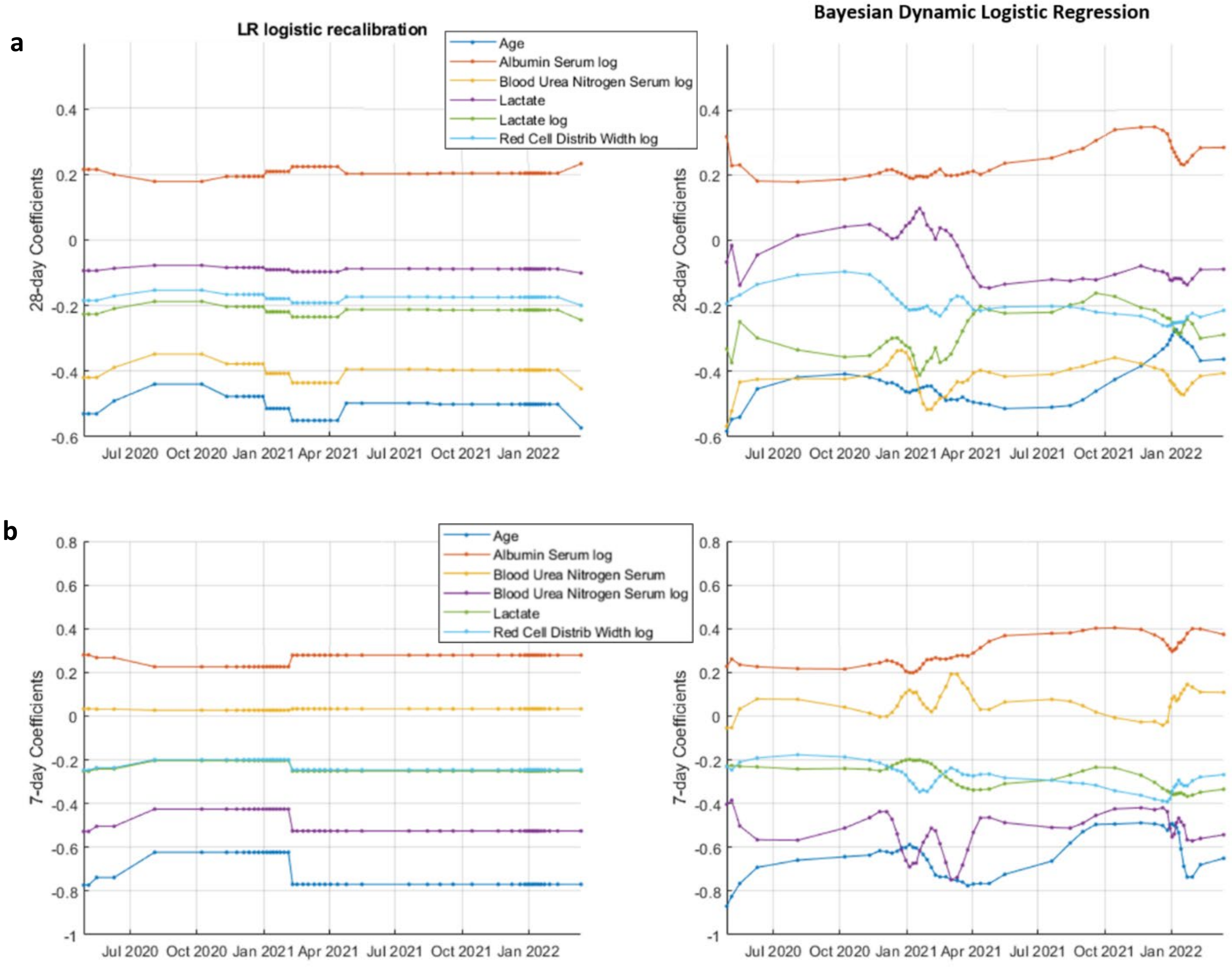

Supplementary Figure 5. **Model coefficient importance. a** predictor importance (without calibration gain and offset) for 7-day NOCOS, **b** logistic regression, and **c** XGBoost models. The importance of the NOCOS and logistic regression model coefficients are the coefficients of the linear predictor scaled by the standard deviations of the predictors from the development cohort. The importance of the XGBoost model coefficients is the weighted average over the ensemble of trees of the difference in node risk between the parent and child nodes due to splitting at each predictor.

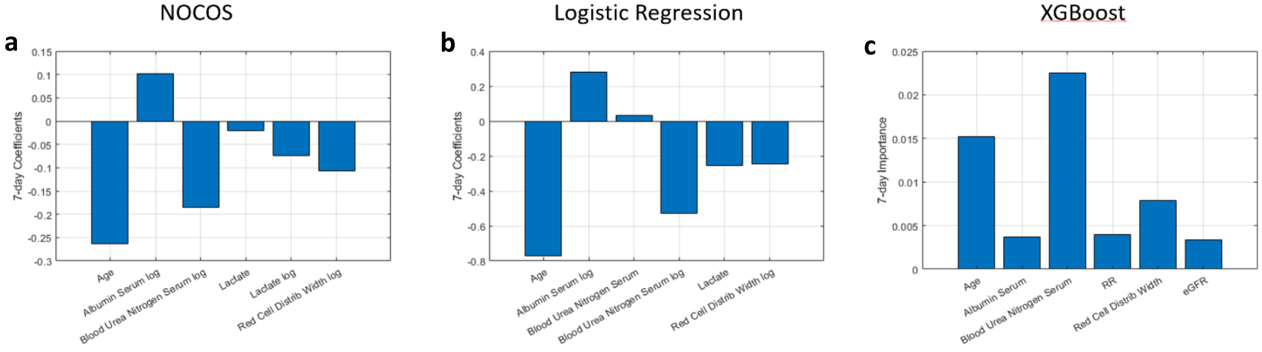

Supplementary Figure 6. **Hyperparameter optimization and causal model design.** Hyperparameter optimization for **a** the number of imputations, **b** ICI update thresholds, and **c** window sizes. **d** Measured optimism bias when assuming that the outcome is known prior to the follow-up period. The baseline for comparison is the 5-imputation, causal NOCOS model with an ICI threshold of 0.03 and a window size of 2000 patients evaluated on the prospective data with patients admitted no later than November 15, 2021 (n=18407). The x-values of the plots have been offset from the labeled x-ticks to prevent overlapping 95% confidence intervals.

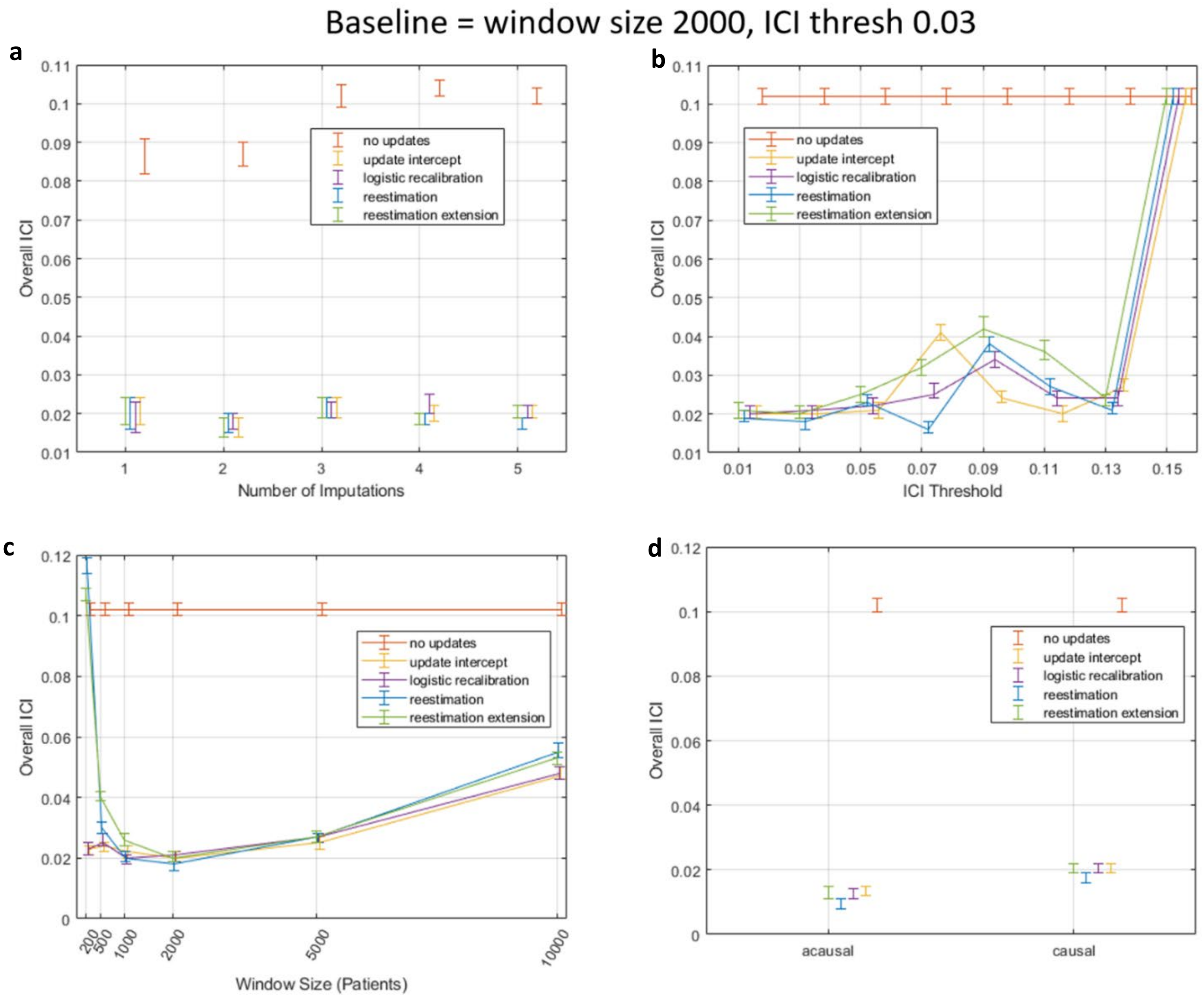

Supplementary Figure 7. **Sensitivity analysis of the 7-day updating NOCOS model across variants, sex and race/ethnicity.** **a** ROC and PR curves with AUC and 95% CI for the prospective (n=25677) validation cohort, and **b** their corresponding calibration plots based on the 7-day NOCOS updated with logistic recalibration. The model was filtered by variant, sex, and race/ethnicity. The points on the calibration show the actual proportion of outcomes averaged over deciles of the predicted probabilities. The diagonal black lines indicate perfect calibration. The ICIs along with their 95% CIs are reported. ROC = receiver operating characteristic; PR = precision recall; AUC = area under the ROC or PR curve; CI = confidence interval; ICI = Integrated calibration index.

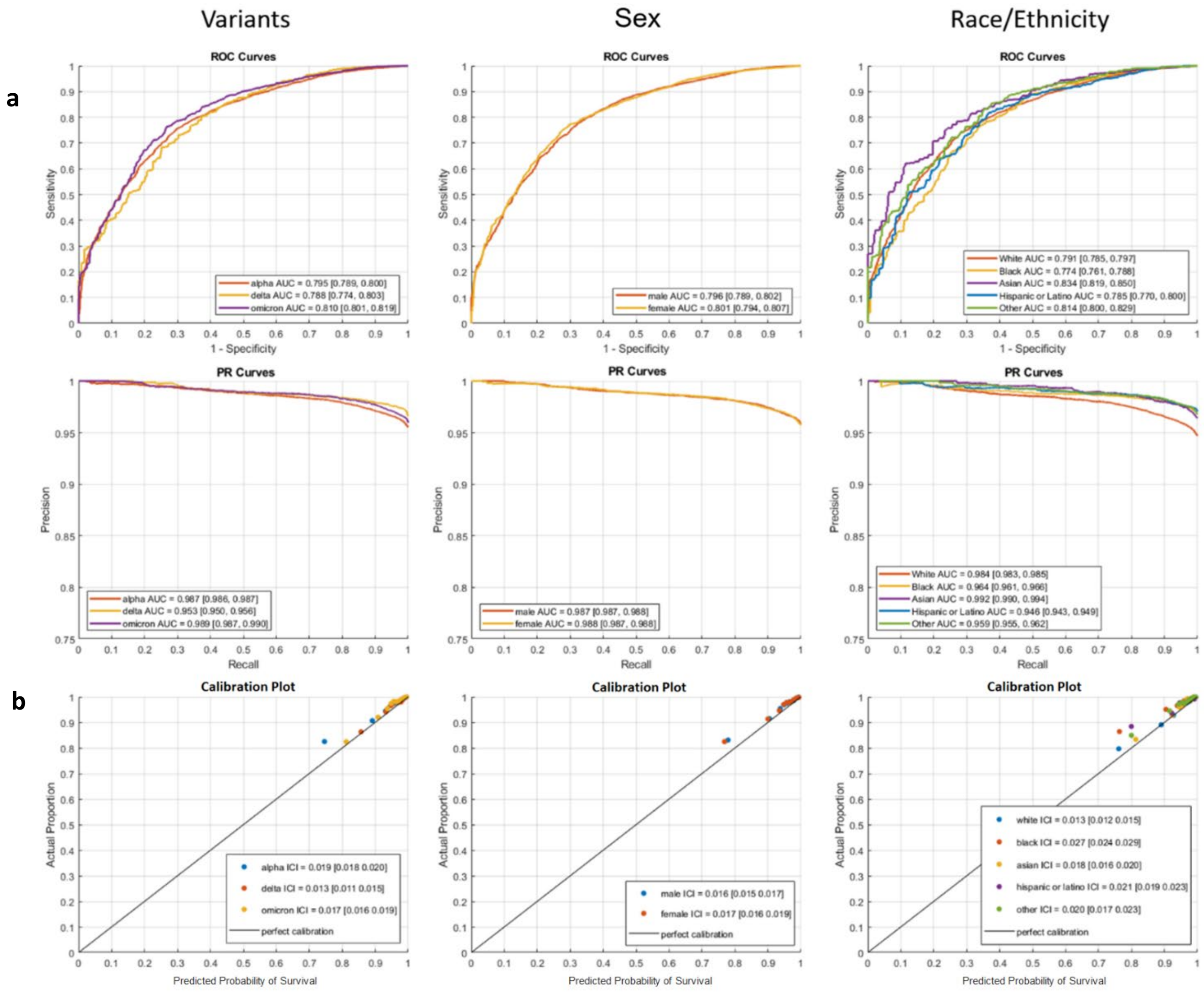

Supplementary Tables

Supplementary Table 1. **Demographic, Clinical, and Laboratory Data of COVID-19 Patients Hospitalized at Northwell Health for 7-day outcomes.** Age is binned in this table for presentation purposes, and the age variable is used as a numeric variable in the models.

	All Included Patients	Alive 7 Days	Died 7 Days	Missing No. (%)
n	34912	32750	2162	
Alpha (% of alpha patients)	25009	23225 (92.9)	1784 (7.1)	0 (0.0)

Delta (% of delta patients)	3475	3358 (96.6)	117 (3.4)	0 (0.0)
Omicron (% of omicron patients)	6428	6167 (95.9)	261 (4.1)	0 (0.0)
Female (%)	15805 (45.3)	14850 (45.3)	955 (44.2)	0 (0.0)
Male (%)	19107 (54.7)	17900 (54.7)	1207 (55.8)	0 (0.0)
Age, y (%)				
18-40 (%)	3434 (9.8)	3397 (10.4)	37 (1.7)	0 (0.0)
41-60 (%)	9618 (27.5)	9409 (28.7)	209 (9.7)	0 (0.0)
61-80 (%)	14746 (42.2)	13825 (42.2)	921 (42.6)	0 (0.0)
81-106 (%)	7114 (20.4)	6119 (18.7)	995 (46.0)	0 (0.0)
Race (%)				
Asian (%)	2818 (8.1)	2648 (8.1)	170 (7.9)	0 (0.0)
Black (%)	6403 (18.3)	6063 (18.5)	340 (15.7)	0 (0.0)
Declined (%)	225 (0.6)	215 (0.7)	10 (0.5)	0 (0.0)
Other (%)	7821 (22.4)	7459 (22.8)	362 (16.7)	0 (0.0)
Unknown (%)	888 (2.5)	840 (2.6)	48 (2.2)	0 (0.0)
White (%)	16757 (48.0)	15525 (47.4)	1232 (57.0)	0 (0.0)
Ethnicity (%)				
Declined (%)	157 (0.5)	151 (0.5)	6 (0.3)	0 (0.0)
Hispanic or Latino (%)	5878 (16.8)	5610 (17.1)	268 (12.4)	0 (0.0)
Not Hispanic or Latino (%)	27516 (78.8)	25707 (78.5)	1809 (83.7)	0 (0.0)
Unknown (%)	1361 (3.9)	1282 (3.9)	79 (3.7)	0 (0.0)
English (%)	29657 (84.9)	27810 (84.9)	1847 (85.4)	0 (0.0)
Length of stay, days (median [IQR])	5.92 [3.15, 11.26]	6.20 [3.33, 11.88]	3.53 [1.91, 5.06]	0 (0.0)
Vented (%)	3713 (10.6)	3184 (9.7)	529 (24.5)	0 (0.0)
Last emergency department vital sign measurement (median [IQR])				
Systolic blood pressure, mmHg	129.00 [115.00, 145.00]	129.00 [115.00, 145.00]	123.00 [109.00, 142.00]	1526 (4.4)
Diastolic blood pressure, mmHg	73.00 [65.00, 82.00]	74.00 [65.00, 82.00]	69.00 [59.00, 78.00]	1526 (4.4)
Heart rate, beats per minute	88.00 [77.00, 100.00]	88.00 [77.00, 100.00]	91.00 [78.00, 105.00]	1509 (4.3)

Respiratory rate, breaths per minute	20.00 [18.00, 22.00]	19.00 [18.00, 22.00]	20.00 [18.00, 25.00]	1589 (4.6)
Temperature, Celsius	37.10 [36.70, 37.70]	37.10 [36.70, 37.70]	37.10 [36.70, 37.90]	1889 (5.4)
Oxygen saturation, %	97.00 [95.00, 99.00]	97.00 [95.00, 99.00]	96.00 [94.00, 99.00]	1594 (4.6)
Body mass index, kg/m2	27.80 [24.30, 32.30]	27.90 [24.40, 32.30]	25.80 [22.30, 30.70]	15643 (44.8)
Height, cm	167.64 [160.02, 175.26]	167.64 [160.02, 175.26]	167.64 [160.02, 172.72]	10765 (30.8)
Weight, kg	79.40 [68.00, 93.00]	79.40 [68.00, 93.40]	72.60 [60.00, 86.20]	12327 (35.3)
Comorbidities, %				
Coronary artery disease (%)	2672 (7.7)	2417 (7.4)	255 (11.8)	8598 (24.6)
Diabetes (%)	6790 (19.4)	6297 (19.2)	493 (22.8)	8598 (24.6)
Hypertension (%)	12293 (35.2)	11385 (34.8)	908 (42.0)	8598 (24.6)
Heart failure (%)	1484 (4.3)	1280 (3.9)	204 (9.4)	8598 (24.6)
Lung disease (%)	3194 (9.1)	2954 (9.0)	240 (11.1)	8598 (24.6)
Kidney disease (%)	1757 (5.0)	1574 (4.8)	183 (8.5)	8598 (24.6)
Last emergency department laboratory result (median [IQR])				
White blood cell count, K/µL	7.34 [5.40, 10.07]	7.27 [5.37, 9.93]	8.67 [6.07, 12.41]	1851 (5.3)
Absolute neutrophil, No., K/µL	5.51 [3.80, 8.01]	5.43 [3.76, 7.87]	6.94 [4.57, 10.34]	2648 (7.6)
Automated neutrophil, %	76.70 [68.30, 83.40]	76.30 [68.10, 83.00]	81.95 [73.90, 87.40]	2618 (7.5)
Automated lymphocyte, No., K/µL	0.93 [0.62, 1.37]	0.94 [0.64, 1.39]	0.75 [0.49, 1.15]	2644 (7.6)
Automated lymphocyte, %	13.10 [8.10, 19.90]	13.40 [8.40, 20.10]	9.00 [5.20, 14.50]	2617 (7.5)
Automated eosinophil, No., K/µL	0.01 [0.00, 0.05]	0.01 [0.00, 0.06]	0.00 [0.00, 0.02]	2695 (7.7)
Automated eosinophil, %	0.10 [0.00, 0.80]	0.10 [0.00, 0.80]	0.00 [0.00, 0.20]	2637 (7.6)

Automated monocyte, No., K/μL	0.51 [0.34, 0.74]	0.51 [0.34, 0.74]	0.49 [0.30, 0.76]	2644 (7.6)
Automated monocyte, %	7.00 [4.90, 9.60]	7.10 [5.00, 9.60]	5.80 [3.70, 8.50]	2617 (7.5)
Hemoglobin, g/dL	12.90 [11.40, 14.20]	13.00 [11.50, 14.20]	12.00 [10.20, 13.80]	1850 (5.3)
Red cell distribution width, %	13.70 [12.80, 15.00]	13.60 [12.80, 14.80]	14.75 [13.60, 16.50]	1876 (5.4)
Automated platelet count, K/μL	214.00 [163.00, 280.00]	214.00 [164.00, 281.00]	201.00 [146.00, 269.00]	1888 (5.4)
Serum sodium, mmol/L	137.00 [134.00, 139.00]	136.00 [134.00, 139.00]	137.00 [134.00, 142.00]	1879 (5.4)
Serum potassium, mmol/L	4.10 [3.70, 4.50]	4.10 [3.70, 4.50]	4.30 [3.90, 4.90]	2076 (5.9)
Serum chloride, mmol/L	100.00 [97.00, 104.00]	100.00 [97.00, 104.00]	101.00 [96.00, 106.00]	1882 (5.4)
Serum carbon dioxide, mmol/L	24.00 [21.00, 26.00]	24.00 [21.00, 26.00]	23.00 [19.00, 25.00]	1872 (5.4)
Serum blood urea nitrogen, mg/dL	18.00 [12.00, 29.00]	17.00 [12.00, 27.00]	34.00 [21.00, 57.00]	1873 (5.4)
Serum creatinine, mg/dL	1.02 [0.80, 1.44]	1.00 [0.80, 1.40]	1.50 [1.03, 2.60]	1871 (5.4)
eGFR mL/min/1.73m2	70.00 [44.00, 92.00]	72.00 [46.00, 93.00]	40.00 [20.00, 62.00]	2168 (6.2)
Serum glucose, mg/dL	122.00 [104.00, 160.00]	121.00 [104.00, 158.00]	136.00 [110.00, 187.00]	1872 (5.4)
Serum albumin, g/dL	3.50 [3.10, 3.90]	3.50 [3.10, 3.90]	3.10 [2.60, 3.60]	2225 (6.4)
Total serum bilirubin, mg/dL	0.50 [0.30, 0.70]	0.50 [0.30, 0.70]	0.60 [0.40, 0.80]	2229 (6.4)
Serum alkaline phosphatase,U/L	78.00 [61.00, 103.00]	78.00 [61.00, 102.00]	86.00 [66.00, 118.00]	2267 (6.5)
Alanine aminotransferase (ALT/SGPT), U/L	29.00 [18.00, 48.00]	29.00 [18.00, 48.00]	28.00 [18.00, 47.00]	2330 (6.7)
Aspartate aminotransferase	37.00 [25.00, 59.00]	37.00 [24.00, 58.00]	48.00 [30.00, 78.00]	2335 (6.7)

(AST/SGOT), U/L				
Serum C-Reactive Protein, mg/L	8.50 [4.00, 15.12]	8.35 [3.90, 14.82]	12.09 [6.21, 20.12]	23730 (68.0)
Serum Lactate	1.70 [1.30, 2.20]	1.60 [1.20, 2.20]	2.10 [1.50, 3.30]	15506 (44.4)

Supplementary Table 2. **7-day model performance metrics for the prospective (n=25677) cohort**.

7-days

AUROC [95% CI]	NOCOS	Logistic Regression	XGBoost
No updates	0.801 [0.796, 0.805]	0.811 [0.807, 0.815]	0.797 [0.792, 0.801]
Intercept only	0.794 [0.789, 0.798]	0.811 [0.807, 0.816]	**0.794 [0.790, 0.799]**
Logistic recal	**0.798 [0.793, 0.803]**	**0.808 [0.803, 0.812]**	0.798 [0.794, 0.803]
reestimation	0.797 [0.793, 0.802]	0.811 [0.807, 0.816]	0.788 [0.783, 0.792]
Reest ext	0.804 [0.800, 0.809]	0.774 [0.769, 0.779]	0.765 [0.760, 0.770]
Dynamic Bayes	N/A	0.813 [0.808, 0.817]	N/A
AUPR [95% CI]	NOCOS	Logistic Regression	XGBoost
No updates	0.988 [0.987, 0.988]	0.989 [0.988, 0.989]	0.917 [0.915, 0.918]
Intercept only	0.987 [0.987, 0.988]	0.989 [0.988, 0.989]	**0.928 [0.927, 0.929]**
Logistic recal	**0.988 [0.987, 0.988]**	**0.988 [0.988, 0.989]**	0.924 [0.922, 0.925]
reestimation	0.956 [0.955, 0.957]	0.989 [0.988, 0.989]	0.986 [0.986, 0.987]
Reest ext	0.964 [0.963, 0.965]	0.987 [0.986, 0.987]	0.916 [0.915, 0.918]
Dynamic Bayes	N/A	0.989 [0.988, 0.989]	N/A
ICI [95% CI]	NOCOS	Logistic Regression	XGBoost
No updates	0.143 [0.142 0.144]	0.076 [0.075 0.077]	0.070 [0.069 0.071]
Intercept only	0.017 [0.016 0.018]	0.018 [0.017 0.019]	**0.006 [0.006 0.007]**
Logistic recal	**0.017 [0.016 0.018]**	**0.012 [0.011 0.013]**	0.015 [0.014 0.016]
reestimation	0.023 [0.022 0.024]	0.010 [0.009 0.011]	0.012 [0.011 0.013]
Reest ext	0.019 [0.018 0.020]	0.016 [0.015 0.017]	0.010 [0.009 0.011]
Dynamic Bayes	N/A	0.011 [0.010 0.012]	N/A

Bold entries indicate the chosen update method for each model based on ICI. AUROC = area under the receiver-operating-characteristic curve; AUPR = area under the precision-recall curve; CI = confidence interval; ICI = integrated calibration index; NOCOS = Northwell COVID-19 Survival Calculator

Supplementary Table 3. **Sensitivity analysis metrics across all models.** 28-day logistic regression, 28-day XGBoost, 7-day NOCOS, 7-day logistic regression, and 7-day XGBoost sensitivity analysis across variants, sex, and race/ethnicity for AUROC, AUPR, and ICI for the prospective (n=25677) cohort. The values are the mean followed by the 95% CIs.

28-day Logistic Regression	No Update AUROC	Log Recal AUROC	No Update AUPR	Log Recal AUPR	No Update ICI	Log Recal ICI
Variants						

Alpha	0.762 [0.758, 0.767]	0.748 [0.744, 0.753]	0.957 [0.955, 0.958]	0.954 [0.952, 0.955]	0.101 [0.099 0.103]	0.021 [0.020 0.023]
Delta	0.753 [0.743, 0.763]	0.753 [0.743, 0.763]	0.964 [0.961, 0.967]	0.964 [0.961, 0.967]	0.104 [0.100 0.108]	0.020 [0.017 0.024]
Omicron	0.771 [0.764, 0.779]	0.771 [0.764, 0.779]	0.971 [0.969, 0.972]	0.971 [0.969, 0.972]	0.161 [0.157 0.164]	0.019 [0.016 0.020]
Sex						
Male	0.756 [0.752, 0.761]	0.751 [0.746, 0.756]	0.957 [0.955, 0.958]	0.956 [0.954, 0.957]	0.108 [0.106 0.110]	0.016 [0.014 0.018]
Female	0.763 [0.757, 0.768]	0.764 [0.758, 0.769]	0.965 [0.963, 0.966]	0.965 [0.963, 0.966]	0.125 [0.123 0.127]	0.021 [0.019 0.023]
Race/Ethnicity						
Asian	0.778 [0.766, 0.790]	0.771 [0.759, 0.783]	0.964 [0.960, 0.967]	0.963 [0.959, 0.966]	0.088 [0.082 0.093]	0.031 [0.027 0.036]
Black	0.771 [0.762, 0.779]	0.773 [0.764, 0.781]	0.964 [0.962, 0.966]	0.965 [0.962, 0.967]	0.112 [0.108 0.116]	0.020 [0.017 0.022]
Hispanic/Latino	0.773 [0.763, 0.783]	0.764 [0.754, 0.774]	0.971 [0.969, 0.974]	0.970 [0.967, 0.972]	0.091 [0.087 0.095]	0.018 [0.015 0.021]
White	0.746 [0.741, 0.751]	0.744 [0.739, 0.749]	0.953 [0.951, 0.955]	0.953 [0.951, 0.954]	0.133 [0.130 0.135]	0.019 [0.017 0.021]
Other	0.753 [0.742, 0.765]	0.746 [0.735, 0.758]	0.965 [0.962, 0.968]	0.964 [0.961, 0.967]	0.102 [0.097 0.107]	0.024 [0.021 0.028]
28-day XGBoost	No Update AUROC	Intercept Only AUROC	No Update AUPR	Intercept Only AUPR	No Update ICI	Intercept Only ICI
Variants						
Alpha	0.767 [0.763, 0.771]	0.755 [0.751, 0.759]	0.944 [0.943, 0.946]	0.951 [0.950, 0.953]	0.097 [0.095 0.099]	0.018 [0.016 0.020]
Delta	0.761 [0.751, 0.771]	0.761 [0.751, 0.771]	0.946 [0.943, 0.950]	0.946 [0.943, 0.950]	0.104 [0.100 0.108]	0.023 [0.020 0.027]
Omicron	0.757 [0.750, 0.765]	0.759 [0.751, 0.767]	0.955 [0.953, 0.957]	0.966 [0.964, 0.968]	0.145 [0.142 0.148]	0.021 [0.018 0.023]
Sex						
Male	0.759 [0.755, 0.764]	0.754 [0.749, 0.758]	0.943 [0.942, 0.945]	0.955 [0.953, 0.957]	0.108 [0.105 0.110]	0.015 [0.013 0.017]

Female	0.765 [0.760, 0.770]	0.765 [0.760, 0.770]	0.950 [0.948, 0.952]	0.963 [0.962, 0.965]	0.112 [0.110 0.114]	0.018 [0.016 0.020]
Race/Ethnicity						
Asian	0.791 [0.779, 0.802]	0.785 [0.773, 0.797]	0.954 [0.949, 0.958]	0.965 [0.961, 0.968]	0.095 [0.090 0.101]	0.023 [0.020 0.028]
Black	0.762 [0.754, 0.771]	0.766 [0.757, 0.774]	0.950 [0.947, 0.953]	0.962 [0.960, 0.965]	0.108 [0.104 0.112]	0.025 [0.022 0.028]
Hispanic/Latino	0.772 [0.762, 0.781]	0.767 [0.757, 0.776]	0.942 [0.938, 0.945]	0.969 [0.966, 0.971]	0.089 [0.086 0.093]	0.017 [0.014 0.020]
White	0.751 [0.746, 0.756]	0.747 [0.742, 0.752]	0.945 [0.943, 0.946]	0.952 [0.950, 0.953]	0.120 [0.118 0.123]	0.016 [0.014 0.018]
Other	0.761 [0.751, 0.772]	0.755 [0.744, 0.766]	0.947 [0.943, 0.950]	0.962 [0.959, 0.965]	0.102 [0.097 0.107]	0.023 [0.019 0.026]
7-day NOCOS	No Update AUROC	Log Recal AUROC	No Update AUPR	Log Recal AUPR	No Update ICI	Log Recal ICI
Variants						
Alpha	0.801 [0.795, 0.806]	0.795 [0.789, 0.800]	0.987 [0.986, 0.988]	0.987 [0.986, 0.987]	0.139 [0.137 0.140]	0.019 [0.018 0.020]
Delta	0.788 [0.774, 0.803]	0.788 [0.774, 0.803]	0.953 [0.950, 0.956]	0.953 [0.950, 0.956]	0.133 [0.130 0.135]	0.013 [0.011 0.015]
Omicron	0.810 [0.801, 0.819]	0.810 [0.801, 0.819]	0.989 [0.987, 0.990]	0.989 [0.987, 0.990]	0.160 [0.157 0.162]	0.017 [0.016 0.019]
Sex						
Male	0.801 [0.795, 0.807]	0.796 [0.789, 0.802]	0.988 [0.987, 0.988]	0.987 [0.987, 0.988]	0.141 [0.140 0.143]	0.016 [0.015 0.017]
Female	0.801 [0.794, 0.807]	0.801 [0.794, 0.807]	0.988 [0.987, 0.988]	0.988 [0.987, 0.988]	0.145 [0.143 0.147]	0.017 [0.016 0.019]
Race/Ethnicity						
Asian	0.829 [0.814, 0.845]	0.834 [0.819, 0.850]	0.992 [0.990, 0.993]	0.992 [0.990, 0.994]	0.130 [0.126 0.134]	0.018 [0.016 0.020]
Black	0.770 [0.756, 0.784]	0.774 [0.761, 0.788]	0.961 [0.959, 0.964]	0.964 [0.961, 0.966]	0.148 [0.146 0.151]	0.027 [0.024 0.029]
Hispanic/Latino	0.798 [0.783, 0.812]	0.785 [0.770, 0.800]	0.937 [0.934, 0.940]	0.946 [0.943, 0.949]	0.123 [0.120 0.125]	0.021 [0.019 0.023]
White	0.794 [0.788, 0.800]	0.791 [0.785, 0.797]	0.984 [0.983, 0.985]	0.984 [0.983, 0.985]	0.152 [0.150 0.154]	0.013 [0.012 0.015]

Other	0.808 [0.793, 0.823]	0.814 [0.800, 0.829]	0.956 [0.952, 0.959]	0.959 [0.955, 0.962]	0.133 [0.130 0.137]	0.020 [0.017 0.023]
7-day Logistic Regression	No Update AUROC	Log Recal AUROC	No Update AUPR	Log Recal AUPR	No Update ICI	Log Recal ICI
Variants						
Alpha	0.813 [0.807, 0.818]	0.808 [0.803, 0.814]	0.988 [0.987, 0.989]	0.987 [0.987, 0.988]	0.071 [0.069 0.072]	0.018 [0.017 0.020]
Delta	0.811 [0.798, 0.825]	0.811 [0.798, 0.825]	0.991 [0.990, 0.993]	0.991 [0.990, 0.993]	0.068 [0.065 0.071]	0.009 [0.008 0.011]
Omicron	0.810 [0.801, 0.819]	0.810 [0.801, 0.819]	0.989 [0.988, 0.990]	0.989 [0.988, 0.990]	0.094 [0.092 0.096]	0.011 [0.009 0.012]
Sex						
Male	0.814 [0.808, 0.820]	0.805 [0.799, 0.812]	0.989 [0.988, 0.990]	0.988 [0.987, 0.989]	0.073 [0.072 0.074]	0.012 [0.010 0.013]
Female	0.808 [0.802, 0.815]	0.810 [0.804, 0.817]	0.988 [0.987, 0.989]	0.988 [0.987, 0.989]	0.079 [0.078 0.081]	0.013 [0.012 0.015]
Race/Ethnicity						
Asian	0.837 [0.822, 0.853]	0.839 [0.824, 0.854]	0.992 [0.990, 0.994]	0.992 [0.990, 0.994]	0.060 [0.056 0.064]	0.013 [0.011 0.015]
Black	0.796 [0.783, 0.809]	0.794 [0.782, 0.807]	0.990 [0.989, 0.991]	0.990 [0.989, 0.991]	0.078 [0.075 0.080]	0.020 [0.018 0.022]
Hispanic/Latino	0.804 [0.790, 0.818]	0.792 [0.778, 0.807]	0.991 [0.990, 0.992]	0.990 [0.989, 0.992]	0.056 [0.053 0.058]	0.015 [0.013 0.017]
White	0.801 [0.795, 0.807]	0.799 [0.793, 0.805]	0.985 [0.984, 0.986]	0.985 [0.984, 0.986]	0.087 [0.085 0.089]	0.011 [0.009 0.012]
Other	0.820 [0.806, 0.834]	0.811 [0.796, 0.825]	0.992 [0.990, 0.993]	0.991 [0.990, 0.993]	0.065 [0.062 0.068]	0.016 [0.014 0.018]
7-day XGBoost	No Update AUROC	Intercept Only AUROC	No Update AUPR	Intercept Only AUPR	No Update ICI	Intercept Only ICI
Variants						
Alpha	0.802 [0.797, 0.808]	0.797 [0.792, 0.803]	0.915 [0.913, 0.917]	0.933 [0.931, 0.935]	0.066 [0.064 0.067]	0.009 [0.008 0.010]
Delta	0.801 [0.787, 0.814]	0.801 [0.787, 0.814]	0.899 [0.895, 0.904]	0.899 [0.895, 0.904]	0.067 [0.064 0.070]	0.010 [0.008 0.011]

Omicron	0.780 [0.770, 0.790]	0.780 [0.770, 0.790]	0.932 [0.929, 0.934]	0.932 [0.929, 0.934]	0.083 [0.081 0.086]	0.008 [0.007 0.010]
Sex						
Male	0.795 [0.789, 0.802]	0.790 [0.784, 0.797]	0.907 [0.905, 0.909]	0.919 [0.917, 0.921]	0.067 [0.065 0.068]	0.007 [0.006 0.008]
Female	0.799 [0.792, 0.805]	0.800 [0.793, 0.806]	0.928 [0.926, 0.931]	0.938 [0.936, 0.940]	0.074 [0.072 0.075]	0.008 [0.007 0.009]
Race/Ethnicity						
Asian	0.817 [0.801, 0.834]	0.828 [0.812, 0.843]	0.895 [0.889, 0.901]	0.909 [0.904, 0.915]	0.059 [0.055 0.063]	0.014 [0.012 0.016]
Black	0.768 [0.754, 0.782]	0.774 [0.760, 0.788]	0.946 [0.943, 0.949]	0.953 [0.950, 0.956]	0.080 [0.077 0.082]	0.016 [0.014 0.018]
Hispanic/Latino	0.798 [0.784, 0.812]	0.783 [0.768, 0.798]	0.868 [0.863, 0.873]	0.889 [0.885, 0.894]	0.057 [0.054 0.059]	0.013 [0.012 0.015]
White	0.790 [0.783, 0.796]	0.786 [0.780, 0.793]	0.927 [0.925, 0.929]	0.935 [0.933, 0.937]	0.075 [0.073 0.077]	0.009 [0.008 0.010]
Other	0.800 [0.784, 0.815]	0.805 [0.790, 0.820]	0.908 [0.903, 0.912]	0.919 [0.914, 0.923]	0.062 [0.059 0.065]	0.012 [0.010 0.014]

AUROC = area under the receiver-operating-characteristic curve; AUPR = area under the precision-recall curve; ICI = integrated calibration index; IQR = interquartile range; NOCOS = Northwell COVID-19 Survival Calculator.

Supplementary Table 4. **28-day NOCOS sensitivity analysis across variants, sex, and race/ethnicity for AUROC, AUPR, and ICI for the prospective (n=25677) cohort**. The values are the mean followed by the 95% CIs.

	No Update AUROC	Log Recal AUROC	No Update AUPR	Log Recal AUPR	No Update ICI	Log Recal ICI
Variants						
Alpha	0.762 [0.758, 0.766]	0.748 [0.743, 0.752]	0.943 [0.942, 0.945]	0.951 [0.950, 0.953]	0.103 [0.101 0.105]	0.022 [0.020 0.023]
Delta	0.752 [0.742, 0.762]	0.751 [0.741, 0.762]	0.947 [0.943, 0.950]	0.954 [0.951, 0.957]	0.107 [0.103 0.112]	0.020 [0.017 0.023]
Omicron	0.771 [0.763, 0.779]	0.772 [0.764, 0.779]	0.953 [0.951, 0.955]	0.970 [0.969, 0.972]	0.164 [0.161 0.167]	0.019 [0.017 0.021]
Sex						
Male	0.756 [0.751, 0.761]	0.750 [0.745, 0.755]	0.946 [0.945, 0.948]	0.955 [0.954, 0.957]	0.110 [0.108 0.113]	0.016 [0.014 0.017]

Female	0.762 [0.757, 0.767]	0.762 [0.757, 0.768]	0.945 [0.943, 0.946]	0.964 [0.963, 0.966]	0.128 [0.126 0.130]	0.022 [0.020 0.024]
Race/Ethnicity						
Asian	0.778 [0.766, 0.790]	0.771 [0.759, 0.783]	0.948 [0.944, 0.953]	0.962 [0.958, 0.965]	0.089 [0.083 0.096]	0.030 [0.025 0.035]
Black	0.770 [0.761, 0.778]	0.771 [0.763, 0.780]	0.949 [0.946, 0.952]	0.964 [0.962, 0.967]	0.114 [0.110 0.118]	0.020 [0.017 0.023]
Hispanic/Latino	0.772 [0.762, 0.781]	0.761 [0.751, 0.771]	0.943 [0.940, 0.946]	0.969 [0.966, 0.971]	0.093 [0.089 0.097]	0.018 [0.015 0.020]
White	0.746 [0.741, 0.751]	0.744 [0.739, 0.749]	0.942 [0.940, 0.944]	0.952 [0.951, 0.954]	0.136 [0.133 0.138]	0.020 [0.018 0.022]
Other	0.753 [0.741, 0.764]	0.744 [0.733, 0.755]	0.950 [0.946, 0.953]	0.962 [0.959, 0.965]	0.104 [0.099 0.109]	0.025 [0.022 0.028]

AUROC = area under the receiver-operating-characteristic curve; AUPR = area under the precision-recall curve; ICI = integrated calibration index; NOCOS=Northwell COVID-19 Survival Calculator; Log Recal = logistic recalibration.

Supplementary Table 5. **Leave-one-hospital-out retrospective cross-validation.** Mean AUROC, AUPR, and ICI for the retrospective validation cohorts.

Hospital	FHH	FRK	GC	HNT	LHH	LIJ	NSUH	PLV	SIUH	SIUHS	SSH	SY
COVID-19 Patients in retrospective cohort	845	499	322	575	923	1889	1699	392	934	211	820	126
AUROC												
28-day NOCOS	0.747	0.739	0.781	0.767	0.801	0.772	0.75	0.728	0.757	0.725	0.732	0.87
7-day NOCOS	0.824	0.752	0.857	0.854	0.83	0.797	0.818	0.786	0.818	0.719	0.799	0.883
28-day LR	0.8	0.738	0.779	0.767	0.803	0.772	0.748	0.727	0.76	0.724	0.726	0.871
7-day LR	0.814	0.751	0.853	0.854	0.834	0.832	0.812	0.789	0.824	0.716	0.798	0.886
28-day XGBoost	0.801	0.743	0.764	0.762	0.816	0.767	0.753	0.732	0.752	0.728	0.722	0.878
7-day XGBoost	0.826	0.766	0.833	0.852	0.824	0.818	0.813	0.801	0.805	0.692	0.785	0.852
AUPR												
28-day NOCOS	0.845	0.846	0.934	0.936	0.946	0.912	0.896	0.862	0.879	0.818	0.878	0.904
7-day NOCOS	0.914	0.912	0.967	0.973	0.954	0.953	0.958	0.942	0.926	0.864	0.948	0.952
28-day LR	0.888	0.851	0.934	0.939	0.953	0.922	0.912	0.864	0.893	0.827	0.894	0.903
7-day LR	0.921	0.915	0.974	0.984	0.982	0.978	0.967	0.952	0.968	0.887	0.972	0.957
28-day XGBoost	0.88	0.83	0.926	0.926	0.937	0.909	0.907	0.875	0.884	0.807	0.88	0.911
7-day XGBoost	0.891	0.883	0.919	0.88	0.893	0.894	0.923	0.915	0.931	0.863	0.843	0.909
ICI												
28-day NOCOS	0.119	0.073	0.089	0.087	0.101	0.047	0.042	0.07	0.051	0.124	0.046	0.143
7-day NOCOS	0.093	0.053	0.047	0.062	0.066	0.068	0.027	0.057	0.038	0.095	0.031	0.108
28-day LR	0.063	0.082	0.086	0.079	0.096	0.044	0.032	0.069	0.054	0.124	0.053	0.151
7-day LR	0.105	0.05	0.051	0.061	0.063	0.019	0.023	0.06	0.043	0.097	0.031	0.113
28-day XGBoost	0.064	0.082	0.089	0.08	0.083	0.03	0.048	0.076	0.063	0.135	0.048	0.136
7-day XGBoost	0.112	0.045	0.046	0.049	0.056	0.024	0.04	0.05	0.032	0.1	0.029	0.1

AUROC = area under the receiver-operating-characteristic curve; AUPR = area under the precision-recall curve; ICI = integrated calibration index; LR = logistic regression; FHH = Forest Hills Hospital; FRK = Long Island Jewish Valley Stream; GC = Glen Cove Hospital; HNT = Huntington Hospital; LHH = Lenox Hill Hospital; LIJ = Long Island Jewish Hospital; NSUH = North Shore University Hospital; PLV = Plainview Hospital; SIUH = Staten Island University Hospital North Campus; SIUHS = Staten Island University Hospital South Campus; SSH = South Shore University Hospital; SY = Syosset Hospital.

Supplementary Methods

In these supplementary materials we repeat the application of the proposed framework for the 7-day survival time horizon and include all relevant results here. We also include the comparison of the logistic regression coefficients updated with logistic recalibration and dynamic Bayesian logistic regression.

Original NOCOS model

The original NOCOS model is described in Levy et al.[1] and estimates the probability of survival as follows:

$$P(Y = survive|X = x) = \frac{f_{X|Y=survive}(x)P(Y = survive)}{\sum_{y\in\{survive,\,die\}} f_{X|Y=y}(x)P(Y = y)}$$

Lasso-penalized “linear” regression is used to select predictors. Let x be the linear predictor that is the inner product of the linear regression coefficients and the selected predictor values. The likelihood functions $f_{X|Y=survive}(x)$ and $f_{X|Y=die}(x)$ are estimated by fitting a parametric distribution (Lévy alpha stable) to the class-conditional distribution of linear predictor values for each of the two outcome classes; survive and die. The priors $P(Y = survive)$ and $P(Y = die)$ are calculated as the fraction of each type of outcome. One will also notice that the probability of survival calculation is a form of Bayes theorem.

Like the logistic regression model where the probability of survival is calculated using the inverse link function shown here,

$$P(Y = survive|X = x) = g^{-1}(x) = \frac{1}{1 + e^{-x}}$$

the NOCOS model can theoretically be framed in the same way even if the link function cannot be determined symbolically. Technically speaking, NOCOS is a generalized linear model. While logistic regression is more elegant than NOCOS, NOCOS is comparable in performance and faster computationally.

Supplementary References

1. Levy, T. J. *et al.* Development and Validation of a Survival Calculator for Hospitalized Patients with COVID-19. 2020.04.22.20075416 Preprint at https://doi.org/10.1101/2020.04.22.20075416 (2020).
