# Supplementary material for: Development and validation of self-monitoring auto-updating prognostic models of survival for hospitalized COVID-19 patients
Source: Nat Commun. 2022 Nov 10;13:6812. doi: 10.1038/s41467-022-34646-2 (PMC9648888; doi:10.1038/s41467-022-34646-2)
Supplement: Supplementary file 2 — Reporting Summary [file 41467_2022_34646_MOESM2_ESM.pdf]

nature portfolio

Corresponding author(s): Theodoros P. Zanos

Last updated by author(s): 6/8/2022

Reporting Summary

Nature Portfolio wishes to improve the reproducibility of the work that we publish. This form provides structure for consistency and transparency in reporting. For further information on Nature Portfolio policies, see our Editorial Policies and the Editorial Policy Checklist.

Statistics

For all statistical analyses, confirm that the following items are present in the figure legend, table legend, main text, or Methods section.

n/a	Confirmed	
☐	☒	The exact sample size (*n*) for each experimental group/condition, given as a discrete number and unit of measurement
☐	☒	A statement on whether measurements were taken from distinct samples or whether the same sample was measured repeatedly
☐	☒	The statistical test(s) used AND whether they are one- or two-sided<br>*Only common tests should be described solely by name; describe more complex techniques in the Methods section.*
☐	☒	A description of all covariates tested
☐	☒	A description of any assumptions or corrections, such as tests of normality and adjustment for multiple comparisons
☐	☒	A full description of the statistical parameters including central tendency (e.g. means) or other basic estimates (e.g. regression coefficient) AND variation (e.g. standard deviation) or associated estimates of uncertainty (e.g. confidence intervals)
☐	☒	For null hypothesis testing, the test statistic (e.g. *F*, *t*, *r*) with confidence intervals, effect sizes, degrees of freedom and *P* value noted<br>*Give P values as exact values whenever suitable.*
☐	☒	For Bayesian analysis, information on the choice of priors and Markov chain Monte Carlo settings
☒	☐	For hierarchical and complex designs, identification of the appropriate level for tests and full reporting of outcomes
☒	☐	Estimates of effect sizes (e.g. Cohen's *d*, Pearson's *r*), indicating how they were calculated

Our web collection on statistics for biologists contains articles on many of the points above.

Software and code

Policy information about availability of computer code

Data collection	Sunrise Clinical Manager v18.4, Allscripts, Chicago, IL
Data analysis	Matlab 2021b custom code (https://zenodo.org/badge/latestdoi/480857206) using the Statistics and Machine Learning Toolbox, Optimization Toolbox, and optionally the Matlab Report Generator, RStudio v1.4.1717 (MICE package)

For manuscripts utilizing custom algorithms or software that are central to the research but not yet described in published literature, software must be made available to editors and reviewers. We strongly encourage code deposition in a community repository (e.g. GitHub). See the Nature Portfolio guidelines for submitting code & software for further information.

Data

Policy information about availability of data

All manuscripts must include a data availability statement. This statement should provide the following information, where applicable:

- Accession codes, unique identifiers, or web links for publicly available datasets
- A description of any restrictions on data availability
- For clinical datasets or third party data, please ensure that the statement adheres to our policy

The tabular data that support the findings of this study are available in the synapse.org database (https://doi.org/10.7303/syn39792658.1).

Human research participants

Policy information about studies involving human research participants and Sex and Gender in Research.

Reporting on sex and gender	The electronic health records used contain a "Gender" field that is self reported and refers to the biological "Sex". We report the number of males and females included in this study and their respective outcomes in Table 1. We report a sensitivity analysis in Figure 6 and Supplemental Figure 7 that report on the difference in model performance between males and females.
Population characteristics	We provide the covariate-relevant population characteristics in Table 1.
Recruitment	This was a retrospective study and patients were selected for this study if they were admitted to one of the 12 acute care facilities with a positive COVID-19 diagnosis and didn't meet any of the exclusion criteria mentioned in the methods section.<br><br>Direct recruitment not applicable for this project which was approved by the Northwell Health IRB under an exemption pathway (45 CFR 46.104 (d) (4)(iii)) with a waiver of HIPAA authorization for the collection and use of retrospective electronic medical record data to create the models.<br><br>Self-selection bias: decision to show up to the hospital; only including hospital patients. There's selection bias from the Hospital – they chose who to admit and who to send home. These are consistent with clinical practice for admission across the US and consistent with with all clinical studies that look at in-hospital patients.<br><br>Bias is also dynamic but our dynamic modeling accounts for this.
Ethics oversight	Data used in this study were collected under an exemption by the Institutional Review Boards at Northwell Health, exemption category 45 CFR 46.104 (d) (4)(iii).

Note that full information on the approval of the study protocol must also be provided in the manuscript.

Field-specific reporting

Please select the one below that is the best fit for your research. If you are not sure, read the appropriate sections before making your selection.

☒ Life sciences ☐ Behavioural & social sciences ☐ Ecological, evolutionary & environmental sciences

For a reference copy of the document with all sections, see nature.com/documents/nr-reporting-summary-flat.pdf

Life sciences study design

All studies must disclose on these points even when the disclosure is negative.

Sample size	The minimum sample size of both the development and validation cohorts was determined by Riley et al., 2020. The optimal window size for prospective analysis was determined via sensitivity analysis in Supplemental Figure 6. The development sample consisted of COVID-19 patients admitted to any of 11 acute care facilities in the Northwell Health system between March 1, 2020 and April 23, 2020. A twelfth acute care facility was held out for retrospective validation. COVID-19 patients admitted after April 23, 2020 and before April 3, 2022 at any of the 12 acute care facilities were part of the prospective update/validation cohort. A total of 34912 patients were included.
Data exclusions	Exclusion criteria include patients transferred to a hospital outside of the health system and their outcomes were unknown, were started on invasive mechanical ventilation prior to admission, or had a do not resuscitate order placed outside of five days of death. These exclusion criteria are designed so that external influences don't affect the outcome.
Replication	We cycled through the hospitals to hold out as a retrospective validation set to ensure that the initial model was robust. We evaluated 3 different model types with 2 variations of the outcome to also show robustness in the system response. All of our attempts to replicate this were successful as outlined in the manuscript.
Randomization	The groups were based on when the patients were admitted to the hospital in this retrospective analysis.
Blinding	Blinding was not relevant since all of the patients were deidentified.

Reporting for specific materials, systems and methods

We require information from authors about some types of materials, experimental systems and methods used in many studies. Here, indicate whether each material, system or method listed is relevant to your study. If you are not sure if a list item applies to your research, read the appropriate section before selecting a response.

Materials & experimental systems

n/a	Involved in the study
☒	☐ Antibodies
☒	☐ Eukaryotic cell lines
☒	☐ Palaeontology and archaeology
☒	☐ Animals and other organisms
☒	☐ Clinical data
☒	☐ Dual use research of concern

Methods

n/a	Involved in the study
☒	☐ ChIP-seq
☒	☐ Flow cytometry
☒	☐ MRI-based neuroimaging
